# Influence of transplant size on the above- and below-ground performance of four contrasting field-grown lettuce cultivars

**DOI:** 10.3389/fpls.2013.00379

**Published:** 2013-09-27

**Authors:** P. J. Kerbiriou, T. J. Stomph, E. T. Lammerts van Bueren, P. C. Struik

**Affiliations:** ^1^Laboratory of Plant Breeding, Department of Plant Sciences, Wageningen URWageningen, Netherlands; ^2^Department of Plant Sciences, Centre for Crop Systems Analysis, Wageningen URWageningen, Netherlands

**Keywords:** lettuce, transplanting, root activity, nutrient use efficiency

## Abstract

**Background and aims:** Modern lettuce cultivars underperform under conditions of variable temporal and spatial resource availability, common in organic or low-input production systems. Information is scarce on the impact of below-ground traits on such resource acquisition and performance of field-grown lettuce; exploring genetic variation in such traits might contribute to strategies to select for robust cultivars, i.e., cultivars that perform well in the field, even under stress.

**Methods:** To investigate the impact of below-ground (root development and resource capture) on above-ground (shoot weight, leaf area) traits, different combinations of shoot and root growth were created using transplants of different sizes in three field experiments. Genetic variation in morphological and physiological below- and above-ground responses to different types of transplant shocks was assessed using four cultivars.

**Results:** Transplanting over-developed seedlings did not affect final yield of any of the four cultivars. Small transplant size persistently impacted growth and delayed maturity. The cultivars with overall larger root weights and rooting depth, “Matilda” and “Pronto,” displayed a slightly higher growth rate in the linear phase leading to better yields than “Mariska” which had a smaller root system and a slower linear growth despite a higher maximal exponential growth rate. “Nadine,” which had the highest physiological nitrogen-use efficiency (g dry matter produced per g N accumulated in the head) among the four cultivars used in these trials, gave most stable yields over seasons and trial locations.

**Conclusions:** Robustness was conferred by a large root system exploring deep soil layers. Additional root proliferation generally correlates with improved nitrate capture in a soil layer and cultivars with a larger root system may therefore perform better in harsh environmental conditions; increased nitrogen use efficiency can also confer robustness at low cost for the plant, and secure stable yields under a wide range of growing conditions.

## Introduction

In organic or low-input production systems, nutrient availability is more dependent on the soil's biological, chemical, and physical processes that influence mineralization of organic fertilizers than in conventional, high-external input production systems. Indeed, in conventional systems fertilization is provided in a mineral form and nutrients are therefore readily available for uptake by the plants once they are sown or transplanted. In lettuce, the impact of variable temporal or spatial shortage of water and nutrients common in organic production systems may significantly reduce final yields, as shown by Kerbiriou et al. (2013). In lettuce, like in other crop plants, breeding has mainly focused on aboveground characteristics, and modern cultivars have been bred for high-input production systems; these cultivars are characterized by large heads and small root systems (Johnson et al., 2000). The small root systems perform sufficiently in such intensive systems.

Current cultivars also have a shallow root system, concentrated in the top 0.20 m of the soil profile (Johnson et al., 2000) which limits the access to deeper soil zones rich in water and nutrients that have leached through the profile. This root morphotype can affect shoot performance under organic conditions, which entail high temporal and spatial variability of resources availability. Exploring the impact of morphological (e.g., spatial configuration) and physiological (e.g., resource capture efficiency) root traits on shoot growth of lettuce may thus be interesting when evaluating the field performance of cultivars under organic conditions. Such investigation might be valuable in breeding programmes, as a mean to select genotypes with desirable root traits increasing tolerance to abiotic stresses and consequently improved yield stability (Bengough et al., 2006). One way to study the impact of below-ground processes—i.e., root growth and resource capture—on shoot growth of lettuce in field conditions is to impact the equilibrium existing between root and shoot growth, by, for instance, altering the root:shoot ratio during the growth. An easy way to manipulate the root:shoot ratio of lettuce during growth is to use different root:shoot ratios at transplanting.

Transplanting is a common horticultural practice, which aims at increasing productivity in horticultural systems. In Western Europe, field-grown lettuce crops are established from transplants raised in compact peat blocks in greenhouses; because seeds germinate faster and more uniformly in peat blocks than in the field, transplanted crops are more competitive toward early weed infestation (Maltais et al., 2008) and provide a more uniform stand, thus facilitating crop scheduling (Cattivello and Danielis, 2008), reducing cropping time and allowing more plantings per year in the same field. However, transplanting induces a major stress in lettuce cultivation: lettuce seedlings in the optimal stage for transplanting (5–7 leaf stage) often suffer from mechanical root pruning (decapitation of the root tip; Biddington and Dearman, 1984) when seedlings are pulled out of the tray. The loss of root tips and root hairs due to root pruning at transplanting disturbs the root:shoot ratio and induces a “recovery phase” during which shoot growth is suppressed until the previous root:shoot ratio is restored (Bar-Tal et al., 1994a).

During this “recovery phase” capture of water (Grossnickle, 2005) and of nutrients (Bar-Tal et al., 1994b) is impaired to levels below requirements. Moreover, there is an imbalance in root and shoot hormones (Overvoorde et al., 2010) and additional assimilates are allocated to the roots to heal root injuries and restore root growth (Bastow Wilson, 1988). Nevertheless, moderate root pruning at transplanting, despite the need for a “recovery phase,” seems to hardly affect final yields: for instance, Bar-Tal et al. (1994a) found that fruit number or total fresh fruit yield were not significantly reduced in tomato plants whose roots were mildly pruned at transplanting, compared with plants whose roots stayed intact at transplanting. In a recent study, Ros et al. (2003) found that 40% root pruning of rice seedlings at transplanting had only a small effect on shoot growth, reducing grain yield and straw dry matter at maturity by a mere 10%. These findings were established for crops like rice, that require a long field growth; it is unclear what the consequences of root pruning could be on a short-cycle crop like lettuce, which is usually harvested within 100 days of field growth (Mou, 2011).

The small or short-lasting effect of root pruning on shoot growth implies that plants are plastic and able to overcome physical damage and adjust to their environment. Plants developed strategies to overcome the loss of root tips and root hairs at transplanting and to compensate for the subsequent impaired resource capture. For instance, Bar-Tal et al. (1994b) found that root pruning in tomato temporarily increased relative growth rate of the pruned roots compared to the intact roots and that nitrogen uptake per unit root volume was larger for plants with pruned root systems than for intact ones. Cattivello and Danielis (2008) showed that chemical root pruning in a selection of vegetables (asparagus, celery, Treviso chicory, fennel, lettuce, and parsley) resulted in a more fibrous and branched root system and had no long-term impact on yield.

In lettuce, the contribution of root traits to field performance has not yet been investigated. It is not clear yet how plastic the plants are in displaying an adaptive response to stresses in the field, and what the contribution is of root morphological (changes in root spatial exploration) or root physiological (resource uptake for instance) traits to shoot development. We used different types of shocks caused by transplanting as a proxy for stress induction. By creating three levels of stress using three growth stages (i.e., differences in root:shoot ratios and in size) at transplanting, we expect to observe different responses in shoot growth that may be explained by below-ground cues, such as root growth and nitrate uptake.

Moreover, breeders assume that there might be considerable genetic variation in the capacity of lettuce plants to recover from transplanting, based on field observations (Velema and Koper, pers. commun.). This suggests that cultivars may develop various strategies below- and above-ground to overcome the disturbance in root:shoot ratio created by transplanting. This study also aims at identifying genetic variation in the physiological below- and above-ground responses to different types of transplant shocks.

## Materials and methods

### Cultivar choice and growing transplants

Four commercial butter head cultivars, “Mariska,” “Matilda,” “Nadine,” and “Pronto,” were chosen. These were known for their robust performance in the field, but also for differences in growth pattern. In a previous pilot study they also showed contrasting rooting patterns (deep vs. superficial) (Den Otter and Lammerts van Bueren, 2007). These cultivars are commonly sold to conventional and organic growers for cropping in spring, summer, and autumn seasons and have been performing consistently over many years (Enza Zaden, pers. commun.).

Seeds used in each of these experiments originated from seed lots produced under the same environmental conditions. Seeds were sown in 4 × 4 × 4 cm organic peat blocks (Jongerius, Houten, Netherlands) after breaking seed dormancy by exposure to 4°C for 24 h. Transplants were raised in a greenhouse with day temperature of 20°C and night temperature of 15°C.

### Experimental design

Three trials were implemented at two different locations: Wageningen (51.97° N, 5.67° E, Netherlands) in spring 2009 and 2010 and Voorst (52.23° N, 6.08° E, Netherlands) in summer 2009. Each trial included three repetitions. The experimental set up was a complete randomized block design, each block consisting of 12 plots featuring all combinations of four cultivars and three transplant sizes.

### Field conditions

For each trial, weather data (air temperature, radiation, rainfall) were recorded daily (Voorst) or hourly (Wageningen) at the nearest weather station (for the Wageningen trials, data were collected from http://www.met.wau.nl/ and for the Voorst trials, data were collected from the on-farm weather station). Soil temperatures were measured at 4–5 depths (0–0.1, 0.1–0.2, 0.2–0.3, 0.3–0.4, and 0.4–0.5 m) using a data logger. Air and soil temperatures recorded during the growing season at Wageningen in spring 2009 were fairly conducive to crop growth, average daily air temperatures ranging from 9.5 to 20°C and average daily soil temperatures at −0.25 m ranging between 10 and 16°C. Rainfall was rather limited during the experiment (Table 1) but there was no drought stress. In contrast, rainfall during the early spring trial at Wageningen in 2010 was abundant, but air temperatures were rather low: during 36 days (i.e., half of the growing period) the daily mean temperature did not exceed 9.5°C. Average daily soil temperatures recorded at −0.25 m ranged between 6 and 15°C during growth, and did not exceed 10°C during the first month of growth. Experiment Voorst 2009 was conducted during late spring under warm weather. The average daily air and soil temperatures at −0.25 m were 16.5 and 17°C, respectively, with air temperatures above 13°C during 85% of the growing period. Soil temperatures at −0.25 m ranged between 15.5 and 20°C. Cumulated degree-days (based on air temperatures), as well as cumulated rainfall and irrigation (in the case of Voorst 2009) at each sampling date for each trial, are shown in Table 1.

**Table 1:** **Planting and harvesting dates and cumulated thermal time (CDD) and rainfall at the three sampling moments for each of the three field trials**.

	**Wageningen 2009**		**Voorst 2009**		**Wageningen 2010**	
**Planting date**	**1 April 2009**		**25 May 2009**		**23 March 2010**	
	**CDD^a^**	**Rainfall (mm)**	**CDD**	**Rainfall (mm)**	**CDD**	**Rainfall (mm)**
Root sampling 1	111	7.3	152	20.0	152	35.5
Root sampling 2	224	21.5	253	77.4	252	60.4
Root sampling 3	325	32.4	420	83.4	347	91.3
Final harvest date	31 May 2009		30 June 2009		31 May 2010	

a Cumulated Degree-Days (°Cd) after planting at sampling date based on air temperature, using a base temperature of 4°C.

### Treatments

Transplanting shocks were used as a proxy for stress induction: seedlings at different growth stages at the moment of transplanting presented different qualities of transplants; three contrasting transplant sizes were obtained by staggered sowings with intervals of 2 weeks. These differences in growth duration before transplanting resulted in intertwined variations in shoot characteristics (number of leaves, and consecutive leaf area) and in root characteristics (root length and mass, not measured at transplanting because of the organic matter in the peat blocks), and associated with the latter also in different levels of damage of the root system at transplanting:

– “Over-Developed” (OD) transplant size: 7–9 leaf-stage, developed root system largely emerging out of the peat block, many roots tips mechanically removed at transplanting, both changing the root:shoot ratio and causing mechanical damage, in addition to the physiological shock of rather large seedlings;
– “Normally Developed” (ND) transplant size: 5-leaf stage, only few roots emerging out of the peat block, some root tips mechanically removed at transplanting, hardly any mechanical damage or root:shoot ratio change;
– “Under-Developed” (UD) transplant size: 3-leaf stage, no visible roots emerging from the peat block except the tap root which was damaged at transplanting; the shock here was mainly the early transplanting of rather small seedlings.

Crop plants raised from these treatments are called “OD plants,” “ND plants,” and “UD plants,” respectively.

In Voorst 2009 damage caused by a hail storm hastened final harvest by approximately 2 weeks, and therefore harvested plants were not fully mature; as UD plants formed heads very late they were not harvested. The final harvest date in the Wageningen trials was determined according to the marketable stage of head maturation for the ND plants. All treatments were harvested at the same date, no matter head maturation stages (which was visually not affected by the treatments at final harvest).

### Field management

All trial fields had been organically managed and were selected for uniform management in the past and for adequate soil structure. They were fertilized prior to transplanting with 100 kg/ha nitrogen, from seaweed pellets (9% N, 3% P, 3% K + 3% MgO, EcoFertiel, EcoStyle, Appelscha, Netherlands). Weeding was done manually every week. Irrigation was only provided at Voorst in 2009: 10 mm water was given 20 days after transplanting.

### Measurements

#### Calculation of thermal time

Cumulated degree days at each sampling date were calculated as the sum, between the date of transplanting and the sampling date, of the degrees above 4°C (base temperature for lettuce), based on an average daily temperature:

$${\text{CDD}}_{\text{sampling}\;x}=\sum_{\text{day 0}}^{\text{sampling date }x}\left[\frac{\left(T_{\text{max}}+T_{\text{min}}\right)}{2}-T_{\text{base}}\right]$$

where *T*_max_ and *T*_min_ correspond to the maximum and to the minimum temperatures recorded on a certain day, respectively.

#### Shoot measurements

Fresh weight, dry weight, total leaf area, and total number of leaves of three plants per plot were assessed weekly. Final harvest took place 6–10 weeks after transplanting depending on trial. For samples taken at final harvest total nitrogen in the head was measured using the Kjeldahl method. Physiological Nitrogen Use Efficiency (NUE, g DM g^−1^ N in head) was calculated based on the head [N] (g N kg^−1^ DM) extracted by the Kjeldahl method: NUE = 1/head [N].

#### Root measurements

**Figure 1:**
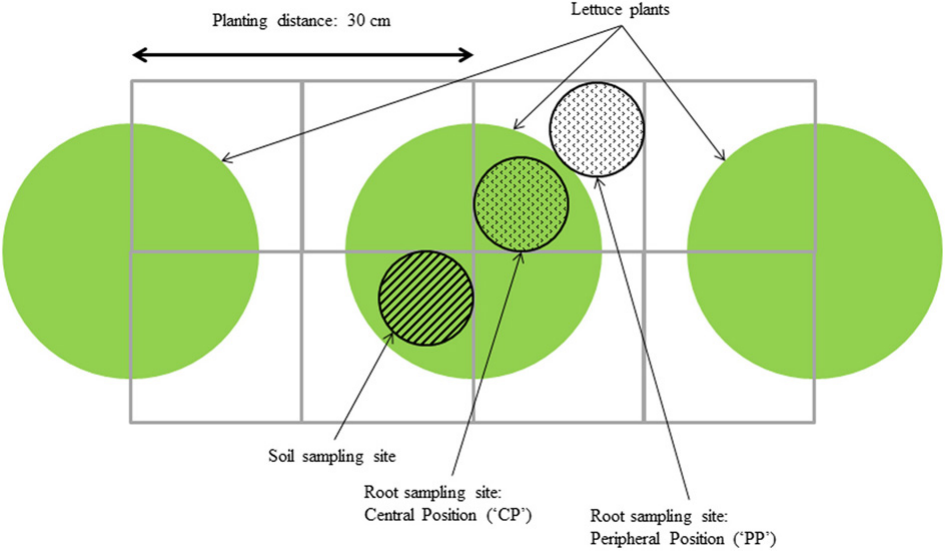
**Root and soil sampling scheme, adapted from Van Noordwijk et al. (1985)**.

Roots outside the peat block of three plants per plot were sampled at three moments during growth, and at two positions (“central” and “peripheral”) for each plant using the method described by Van Noordwijk et al. (1985) (Figure 1). Using a cylindrical auger of 0.07 m diameter and 0.1 m height, samples were taken every 0.1 m over a depth of 0.5 m. For each sample, roots were rinsed from soil and most organic matter using a rinsing machine and remaining organic matter was then manually removed using tweezers. Root samples were subsequently scanned and root length was measured using WinRhizo Pro 2007 (v2005b, Regent Instruments, Québec, Canada). Root dry matter was measured after drying the root samples at 105 °C for 24 h. Root Mass Density per layer (mg root dry weight g^−1^ soil) was calculated as root dry weight measured in the sample taken with the auger, divided by the product of the volume of soil in the sample taken and the bulk density of that soil (based on dry weight).

#### Soil measurements

Soil samples were taken simultaneously on the opposite side of the same plants (Figure 1). For three plants per plot, soil samples were pooled to account for plant-to-plant variation. Soil moisture content was recorded after drying at 40°C for 48 h and soil nitrate content (soil [NO_3_]) was measured using an Ion Selective Electrode (ThermoFisher, Waltham, MA, USA) using the method described previously by Sibley et al. (2009) and also used in Kerbiriou et al. (2013). As a measure for the difference between treatments in estimated NO_3_ capture, the difference between the average soil [NO_3_], based on pooled data for all cultivar × transplant size combinations within a layer, and the soil [NO_3_] measured on an individual plot was expressed as percentage difference in estimated NO_3_ capture. This was calculated as:

% difference for sample *i* = 100 × (([NO_3_]_i_/[NO_3_]_avg_) − 1)

Where

[NO_3_]_*i*_ = observed [NO_3_] in sample *i* on sampling date *d* and for soil layer *l*

[NO_3_]_avg_ = the average observed [NO_3_] in all samples on sampling date *d* and for soil layer *l*.

### Statistical analyses

Dry weight and total leaf area data of all harvests for each trial were pooled per plot and a regression analysis was performed using the expolinear model of Goudriaan and Monteith (1990) to obtain estimates of the curve fit parameters for each combination of transplant size × cultivar × replicate. Then a two-way ANOVA was performed on those parameters to determine main effects of stage at transplanting (UD, ND, and UD), cultivar and their interactions, followed by a Tukey test *p-value* ≤ 0.05 to determine the statistical significance of the differences.

Moreover, for each sampling date for each trial a two-way ANOVA was performed followed by the Tukey test at *p-value* ≤ 0.05 to determine the statistical significance of the differences.

Curve fitting and statistical analyses were performed with Genstat 15th Edition (Hempstead, UK).

## Results

### Effect of transplant size on shoot growth and development

**Figure A1:**
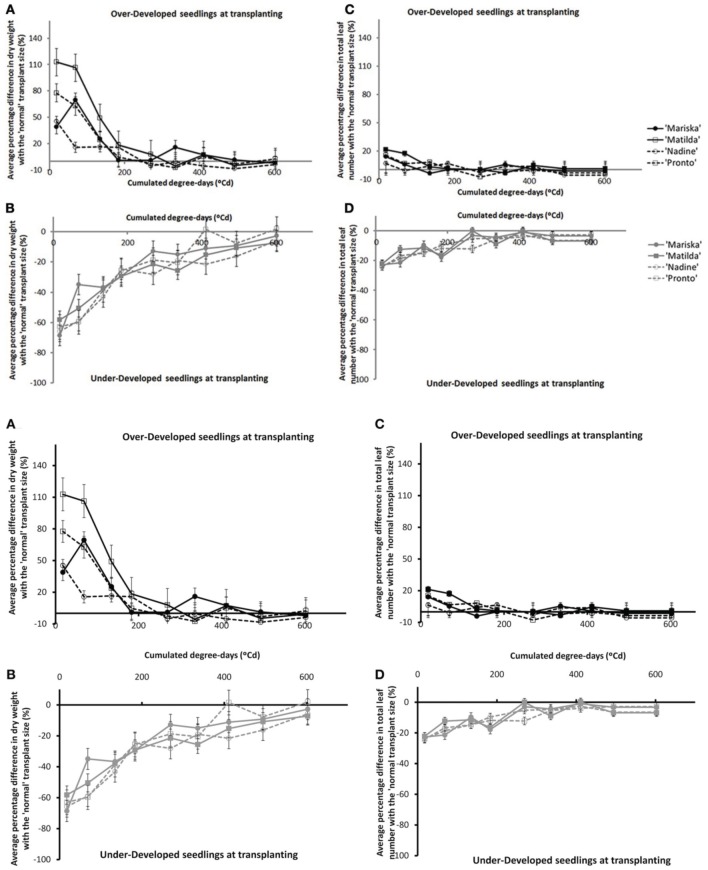
**Average percentage difference in dry weight of plants originating from Over-Developed (A) and Under-Developed (B) seedlings, in comparison with the dry weights of plants originating from Normally Developed seedlings, and average percentage difference in total number of leaves of plants originating from Over-Developed (C) and Under-Developed (D) seedlings in comparison with the number of leaves counted on plants originating from Normally Developed seedlings for the four cultivars (trial Wageningen 2009).** Error bars indicate ± one standard deviation.

The overall effects of transplant size on dry matter accumulation and total number of leaves decreased in time after transplanting (cf. Figure A1). Differences between the Over-Developed- (“OD”) or the Under-Developed (“UD”) plants and the Normally-Developed (“ND”) plants when expressed in percentages were larger for dry matter accumulation than for total number of leaves, and these differences disappeared faster for the OD plants than for the UD plants (cf. Figures A1A, A1B). After 200°Cd there was less than 20% difference in dry matter between the OD and the ND plants, whereas this level was reached by 500°Cd for the UD plants. No cultivar differences were observed. The same trends were observed in all experiments.

#### Dry matter accumulation

**Figure 2:**
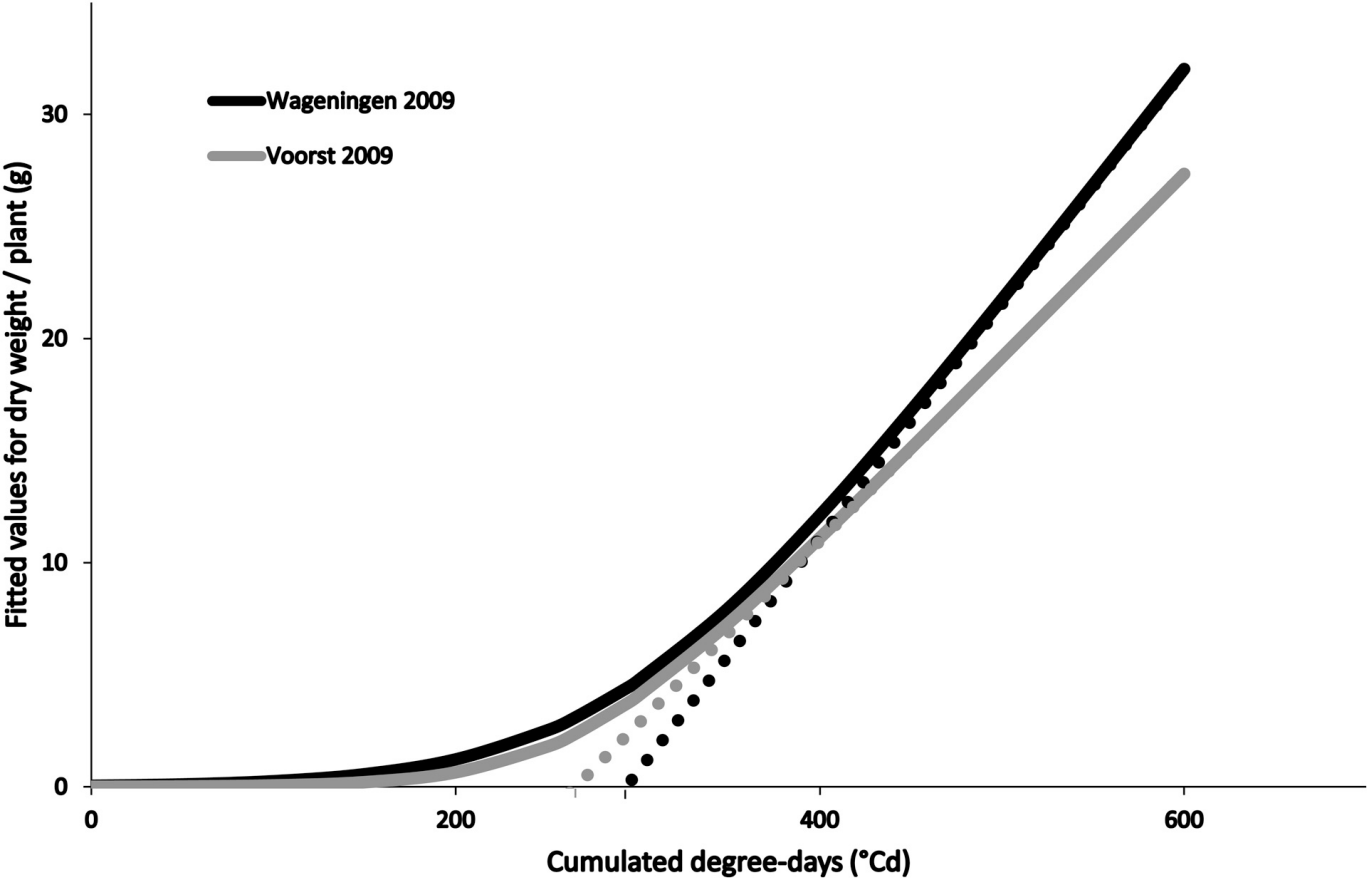
**Fitted values for dry weight accumulation over thermal time (based on average curve parameters for cultivars and transplant sizes within a trial, cf. Table 2) in Wageningen 2009 and Voorst 2009.** The asymptotes to the expolinear curves cut the x-abscissa at the values obtained for “lag phase” which are, in this case, 293°C for Wageningen 2009 and 262°C for Voorst 2009.

Differences in growing conditions affected the dry matter accumulation of the four cultivars, independently of stage at which they were transplanted, although all followed a typical expolinear growth pattern (Goudriaan and Monteith, 1990; Figure 2). Overall warmer growing conditions recorded during Voorst 2009 led to a higher maximal relative growth rate during the initial exponential growth phase, a lower maximal growth rate during the linear growth phase, and a reduced “lag phase” (time at which the asymptote of the expolinear growth curve meets the time abscissa, cf. Figure 2), compared to the trials conducted in Wageningen in 2009 and 2010 (Table 2).

**Table 2:** **Values for curve fit parameters when applying an expolinear model (Goudriaan and Monteith, 1990) for dry matter accumulation against thermal time, for combinations of transplant sizes and cultivars in each of three experiments**.

	**Maximal relative growth rate in the exponential phase (mg g^−1^ (°Cd)^−1^)**					**Maximal growth rate in the linear phase (mg DM m^−2^ (°Cd)^−1^)**					**“Lag phase” (°Cd)**				
**Cultivar:**	**Mariska**	**Matilda**	**Nadine**	**Pronto**		**Mariska**	**Matilda**	**Nadine**	**Pronto**		**Mariska**	**Matilda**	**Nadine**	**Pronto**	
***TS*^f^**	**Wageningen 2009**				***Tr.*^d^**	**Wageningen 2009**				***Tr*.**	**Wageningen 2009**				***Tr*.**
OD^a^	15.9	14.4	15.4	14.7	*15.1*a	91	109	110	112	*106*a	267	306	299	304	*294*ab
ND^b^	17.7	16.5	16.8	16.8	*16.9*b	93	102	113	104	*103*a	265	292	292	283	*283*a
UD^c^	18.8	16.5	16.3	17.9	*17.4*b	93	104	116	106	*105*a	279	318	323	296	*304*b
*Cv.^e^*	*17.5*b^g^	*15.8*a	*16.2*ab	*16.5*ab		*92*a	*105*b	*113*b	*107*b		*271*a	*305*b	*305*b	*294*ab	
	**Wageningen 2010**				***Tr*.**	**Wageningen 2010**				***Tr*.**	**Wageningen 2010**				***Tr*.**
OD	16.0	14.9	14.7	14.9	*15.1*a	103	180	145	140	*142*a	255	311	291	286	*286*a
ND	16.5	18.2	17.3	16.8	*17.2b*	132	140	125	130	*132*a	283	273	274	278	*277*a
UD	16.7	19.3	19.4	18.6	*18.5b*	126	129	123	113	*123*a	316	319	309	309	*313*b
*Cv*.	*16.4*a	*17.5*a	*17.1*a	*16.7*a		*121*a	*149*a	*131*a	*128*a		*285*a	*301*a	*291*a	*291*a	
	**Voorst 2009**				***Tr*.**	**Voorst 2009**				***Tr*.**	**Voorst 2009**				***Tr*.**
OD	21.1a	18.5a	20.7a	16.5a	*19.2*	68	100	78	101	*87*a	208	267	222	277	*244*a
ND	41.3b	24.8a	25.2a	22.4a	*28.4*	48	105	75	80	*77*a	218	330	303	303	*288*b
UD	–	–	–	–		–	–	–	–		–	–	–	–	
*Cv.*	*31.2*	*21.7*	*23.0*	*19.5*		*58*a	*103*b	*76*ab	*90*b		*213*a	*299*b	*262*b	*290*b	

a “Over-developed” transplant size.

b “Normally developed” transplant size.

c “Under-developed” transplant size.

d Mean for transplant size across cultivars.

e Mean for cultivar across transplant sizes.

f Transplant size.

g Means with different letters indicate a significant difference at p ≤ 0.05—means separation with lettering is indicated for each single parameter within an experiment and at the level of main factors cultivar or transplant size when the two-way interaction was not significant, and at the level of transplant size × cultivar when the interaction was significant.

***Maximal relative growth rate during exponential phase.*** During the exponential growth phase, OD plants had a significantly smaller maximal relative growth rate than ND and UD plants, while no differences were observed between ND and UD in Wageningen 2009 and 2010. In Wageningen 2009 “Mariska” had the highest maximal relative growth rate for all transplant sizes. The two-way interaction was not significant in Wageningen 2009 and 2010, while it was in Voorst 2009. Here the same trend was observed as in the Wageningen trials but only the maximal relative growth rate of “Mariska” ND plants was different from all other treatments.

***Maximal growth rate during the linear phase.*** No significant effect of transplant size was recorded on the maximal growth rate during the linear phase in any of the three trials. “Mariska” had a significantly lower growth rate than the other cultivars in the linear phase for all transplant sizes in Wageningen 2009 and Voorst 2009. The same trend was observed in Wageningen 2010, albeit not significant (*p-value* = 0.058). No two-way interactions were significant.

***“Lag phase”.*** UD plants had a longer lag phase in both Wageningen trials than OD and ND plants. In Voorst 2009, OD plants had a shorter lag phase than ND plants (Table 2). In Wageningen 2009 and Voorst 2009, “Mariska” had a significantly shorter lag phase than other cultivars across transplant sizes (Table 2). No two-way interactions were significant.

***Dry weight at final harvest.*** While there was no significant effect of transplant size on dry weight at final harvest in Wageningen 2009, cultivar differences were visible, with “Mariska” having the lowest dry weight at final harvest and “Nadine” performing the best (Table 3). In Wageningen 2010, significant interactions between transplant size and cultivar effects were recorded. No significant difference at *p* = 0.05 was found between cultivars within the UD and the ND transplant size. OD plants of “Matilda” and “Nadine” had higher final dry weights than OD plants of “Mariska.” Whereas UD plants of “Matilda” and “Pronto” had significantly smaller dry weights at final harvest compared to ND and OD plants of these cultivars, for “Mariska” and “Nadine” there was no significant effect of transplant size on dry weight at final harvest.

**Table 3:** **Average shoot dry weights (g per plant) of the four cultivars at final harvest, after establishment from three different transplant sizes in each of three trials**.

**Harvest Date**	**CDD^f^ (°Cd)**	**TS^h^**	**Mariska**	**Matilda**	**Nadine**	**Pronto**	
			**Wageningen 2009**				***Tr.*^*e*^**
May 25th, 2009	474	OD^a^	30.3 ± 2.3^g^	32.1 ± 3.1	33.9 ± 4.9	33.1 ± 2.0	*32.3*a
		ND^b^	31.0 ± 2.1	32.3 ± 2.1	35.3 ± 2.4	33.0 ± 3.2	*32.7*a
		UD^c^	30.2 ± 2.5	30.0 ± 2.0	33.3 ± 2.7	33.0 ± 2.1	*31.6*a
		*Cv.^d^*	*30.5*a^i^	*31.5*ab	*34.1*c	*32.8*bc	
			**Wageningen 2010**				***Tr*.**
May 30th, 2010	400	OD	25.4 ± 2.2abcde	34.0 ± 4.7g	31.3 ± 2.6fg	29.7 ± 3.5efg	*30.1*
		ND	29.1 ± 2.1cdefgh	33.0 ± 3.8fg	29.4 ± 2.9defg	28.5 ± 3.5bcdef	*30.0*
		UD	23.5 ± 1.8ab	24.0 ± 3.1abc	24.3 ± 3.5abcd	22.4 ± 4.7a	*23.6*
		*Cv*.	*26.0*	*30.4*	*28.3*	*26.9*	
			**Voorst 2009**				***Tr*.**
June 29th, 2009	420	OD	18.5 ± 1.9	22.6 ± 2.7	20.5 ± 2.5	21.5 ± 2.7	*20.8*b
		ND	13.1 ± 2.0	17.5 ± 2.1	14.2 ± 2.2	14.8 ± 3.3	*14.9*a
		UD	–	–	–	–	
		*Cv*.	*15.8*a	*20.1*c	*17.4*ab	*18.2*b	

a “Over-developed” transplant size.

b “Normally developed” transplant size.

c “Under-developed” transplant size.

d Mean for cultivar across transplant sizes.

e Mean for transplant size across cultivars.

f Cumulated Degree-Days.

g Standard error of the mean.

h Transplant Size.

i Means with different letters indicate a significant difference at p ≤ 0.05—means separation with lettering is within an experiment and at the level of main factors cultivar or transplant size when the two-way interaction was not significant and at the level of transplant size × cultivar when the interaction was significant.

In Voorst 2009, OD plant had significantly higher dry weight at final harvest than ND plants (Table 3). “Matilda” had a significantly higher final dry weight per plant than other cultivars across transplant sizes, whereas “Mariska” had the lowest dry weight at final harvest across transplant sizes.

(Shoot dry weights measured at intermediate root samplings are presented in the supplementary materials, Tables S1, S2).

#### Leaf area expansion

Interestingly no significant cultivar effect was found on the curve fit parameters of an expolinear model on leaf area expansion (Table 4). On the other hand, size at transplanting significantly affected the leaf area expansion rates of the plants both during the exponential and the linear growth phases.

**Table 4:** **Values for curve fit parameters when applying an expolinear model (Goudriaan and Monteith, 1990) for leaf area expansion against thermal time**.

	**Maximal relative growth rate in the exponential phase (mm^2^ cm^−2^ (°Cd)^−1^)**					**Maximal leaf expansion rate in the linear phase (cm^2^ m^−2^ (°Cd)^−1^)**					**Time lost during canopy development before all radiation is intercepted (°Cd)**				
**Cultivar:**	**Matilda**	**Mariska**	**Nadine**	**Pronto**		**Matilda**	**Mariska**	**Nadine**	**Pronto**		**Matilda**	**Mariska**	**Nadine**	**Pronto**	
***TS*^f^**	**Wageningen 2009**				***Tr.*^d^**	**Wageningen 2009**				***Tr*.**	**Wageningen 2009**				***Tr*.**
OD^a^	1.46	1.36	1.51	1.36	*1.42*a	40.6	43.1	41.9	45.2	*42.7*b	309	350	330	353	*335*b
ND^b^	1.64	1.65	1.56	1.57	*1.60*b	34.9	27.3	38.5	36.3	*34.2*ab	291	285	321	307	*301*a
UD^c^	1.77	1.67	1.73	1.83	*1.75*c	37.6	24.5	27.9	35.1	*31.3*a	305	301	309	300	*304*a
*Cv.^e^*	*1.62a^g^*	*1.56*a	*1.60*a	*1.59*a		*37.7*a	*31.6*a	*36.1*a	*38.8*a		*301*a	*312*a	*320*a	*320*a	
	**Wageningen 2010**				***Tr*.**	**Wageningen 2010**				***Tr*.**	**Wageningen 2010**				***Tr*.**
OD	1.53	1.58	1.38	1.57	*1.52*a	31.5	40.0	45.5	35.0	*38.0*a	290	304	355	302	*313*a
ND	1.54	1.63	1.56	1.65	*1*.60a	41.8	36.5	36.0	38.2	*38.1*a	321	308	325	308	*316*a
UD	1.83	1.65	1.84	1.70	*1.75*b	30.9	40.0	29.1	35.5	*33.9*a	308	366	329	352	*33*9a
*Cv*.	*1.63*a	*1.62*a	*1.60*a	*1.64*a		*34.7*a	*38.9*a	*36.9*a	*36.2*a		*307*a	*326*a	*336*a	*321*a	

a “Over-developed” transplant size.

b “Normally developed” transplant size.

c “Under-developed” transplant size.

d Mean for transplant size across cultivars.

e Mean for cultivar across transplant size.

f Transplant size.

g Means with different letters indicate a significant difference at p ≤ 0.05—means separation with lettering is indicated for each single parameter within an experiment and only at the level of main factors cultivar and transplant size as the two-way interactions were not significant.

***Maximal relative leaf area expansion rate during the exponential phase.*** In Wageningen 2009 and Wageningen 2010, UD plants of all cultivars had a significantly higher maximal relative leaf expansion rate during the exponential phase than ND and OD plants (Table 4). In Wageningen 2009, OD plants had a significantly lower maximal relative leaf expansion rate during the exponential phase than ND plants (Table 4).

***Maximal leaf area expansion rate during the linear phase.*** In Wageningen 2009, the leaf expansion rate of the UD and ND plants of all cultivars was reduced during the linear phase compared to OD plants (Table 4).

***“Lag phase”.*** A significantly longer lag phase was found for the OD plants of all cultivars compared to ND and UD plants (Table 4) only in Wageningen 2009.

### Effect of transplant size on root growth and resource capture

#### Root dry weights

In Voorst 2009, overall measured root dry weights were much lower than in Wageningen 2009 and Wageningen 2010 due to the precocious termination of the trial (Table 5).

**Table 5:** **Average estimated root dry weights (g per plant) of the four cultivars at third root sampling, after establishment from three different transplant sizes**.

**Harvest Date**	**CDD^f^ (°Cd)**	**TS^h^**	**Mariska**	**Matilda**	**Nadine**	**Pronto**	
			**Wageningen 2009**				***Tr.*^e^**
May 11th, 2009	325	OD^a^	0.39 ± 0.14^g^	0.47 ± 0.27	0.44 ± 0.12	0.48 ± 0.21	*0.44*a
		ND^b^	0.35 ± 0.14	0.55 ± 0.26	0.47 ± 0.18	0.53 ± 0.22	*0.48*a
		UD^c^	0.36 ± 0.10	0.42 ± 0.15	0.46 ± 0.17	0.47 ± 0.17	*0.43*a
		*Cv.^d^*	*0.37*a^i^	*0.48*ab	*0.46*ab	*0.49*b	
			**Wageningen 2010**				***Tr*.**
May 25th, 2010	347	OD	0.61 ± 0.22	0.68 ± 0.12	0.52 ± 0.17	0.67 ± 0.20	*0.62*b
		ND	0.58 ± 0.17	0.63 ± 0.22	0.66 ± 0.32	0.74 ± 0.22	*0.65*b
		UD	0.40 ± 0.07	0.48 ± 0.19	0.57 ± 0.22	0.55 ± 0.17	*0.50*a
		*Cv*.	*0.53*a	*0.60*a	*0.59*a	*0.65*a	
			**Voorst 2009**				***Tr*.**
June 29th, 2009	420	OD	0.18 ± 0.03	0.28 ± 0.06	0.24 ± 0.12	0.29 ± 0.09	*0.25*a
		ND	0.12 ± 0.10	0.24 ± 0.11	0.15 ± 0.05	0.29 ± 0.10	*0.20*a
		UD	–	–	–	–	*–*
		*Cv*.	*0.15*a	*0.26*bc	*0.20*ab	*0.29*c	

a “Over-developed” transplant size.

b “Normally developed” transplant size.

c “Under-developed” transplant size.

d Mean for cultivar across transplant sizes.

e Mean for transplant size across cultivars.

f Cumulated Degree-Days.

g Standard error of the mean.

h Transplant size.

i Means with different letters indicate a significant difference at p ≤ 0.05—means separation with lettering is within an experiment and at the level of main factors cultivar or transplant size as the two-way interaction was not significant.

In Wageningen 2009 and Voorst 2009, no significant transplant size effect was found on root weight at final harvest. On the other hand, significantly lower root weights were observed for all cultivars of UD plants compared to OD- and ND plants in Wageningen 2010 (Table 5). In this trial, no significant cultivar effect was measured, whereas these were recorded in Wageningen 2009 and Voorst 2009. In both trials, “Mariska” had—on average for all transplant sizes—a lower total root weight per plant than “Pronto” (Table 5).

(Root dry weights measured at intermediate root samplings are presented in the supplementary materials, Tables S3, S4).

#### Root mass densities over the soil profile

**Figure 3:**
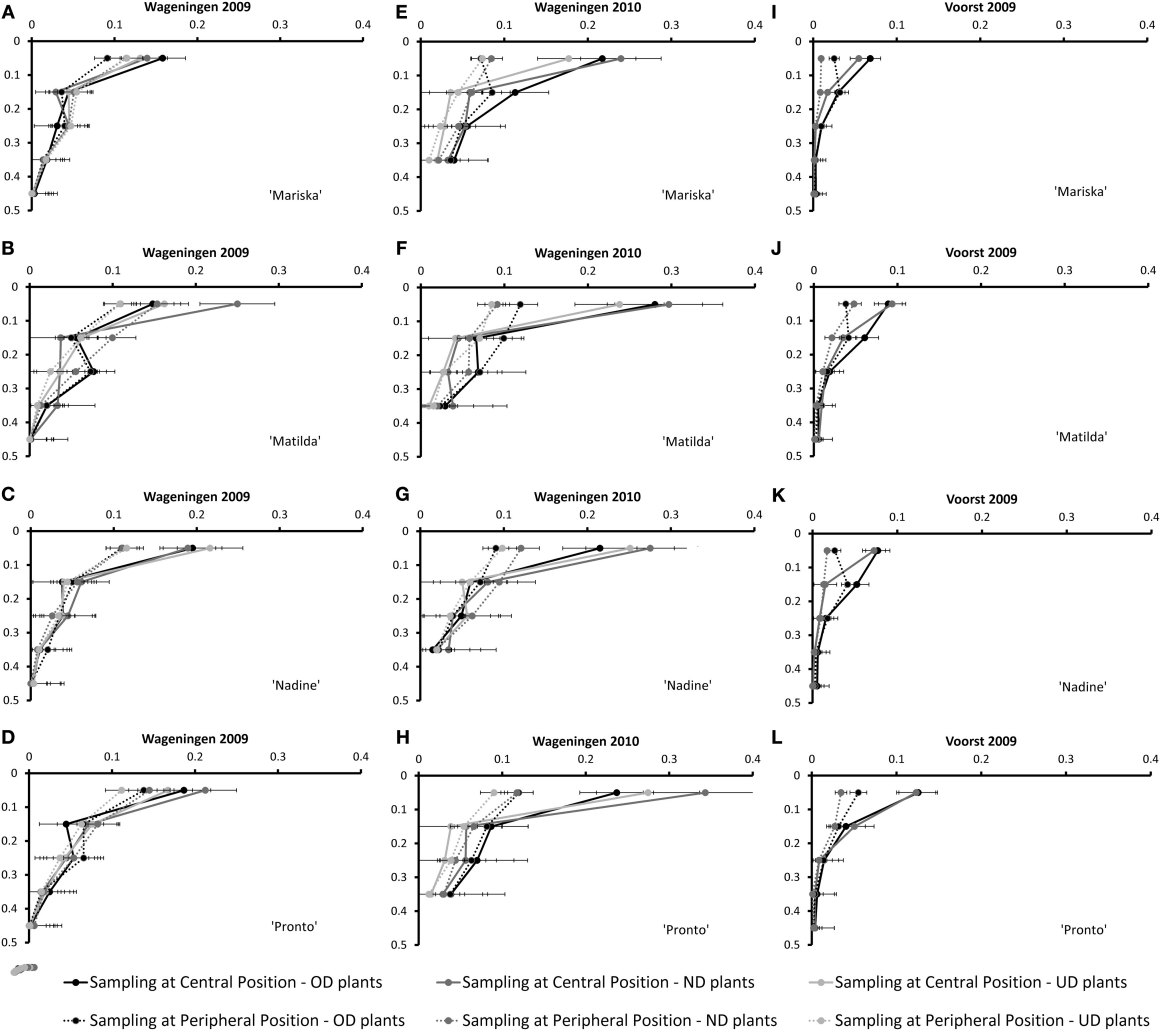
**Root Mass Density measured at the Central- and Peripheral positions at the third root sampling of the four cultivars averaged over the three or two transplant sizes [Over-Developed- (“OD”), Normally Developed- (“ND”) and Under-Developed- (“UD”) transplant size] for the trials Wageningen 2009 (A–D), Wageningen 2010 (E–H) and Voorst (I–L) (for sampling method, cf. Figure 1).** Error bars indicate ± one standard deviation.

Figure 3 shows the root mass densities for the four cultivars under the three transplant sizes over the soil profile at the third root sampling date, both at the central- and at the peripheral sampling position (cf. Figure 1).

Apparently, the most important element of variation in root spatial (horizontal and vertical) exploration (as measured by root mass densities over the soil profile at the different sampling positions) was conferred by the growing season: whereas under the rather optimal conditions in Wageningen 2009 (Figures 3A–D), the root mass density measured in the top 0.1 m at the central sampling position was rather identical to the root mass density measured at the peripheral position for all cultivars, with the exception of “Nadine” (Figure 3C), under the much cooler conditions in Wageningen 2010 a larger root mass density was measured at the central position compared with the peripheral sampling position (Figures 3E–H). The same pattern was observed, although to a lesser extent, under the rather warm conditions in Voorst 2009 (Figures 3I–L). The transplant sizes did not influence the root mass density distribution over the soil profile in any of the three trials.

#### Relationship between NO_3_ capture from the soil and RLD (root length density)

**Figure 4:**
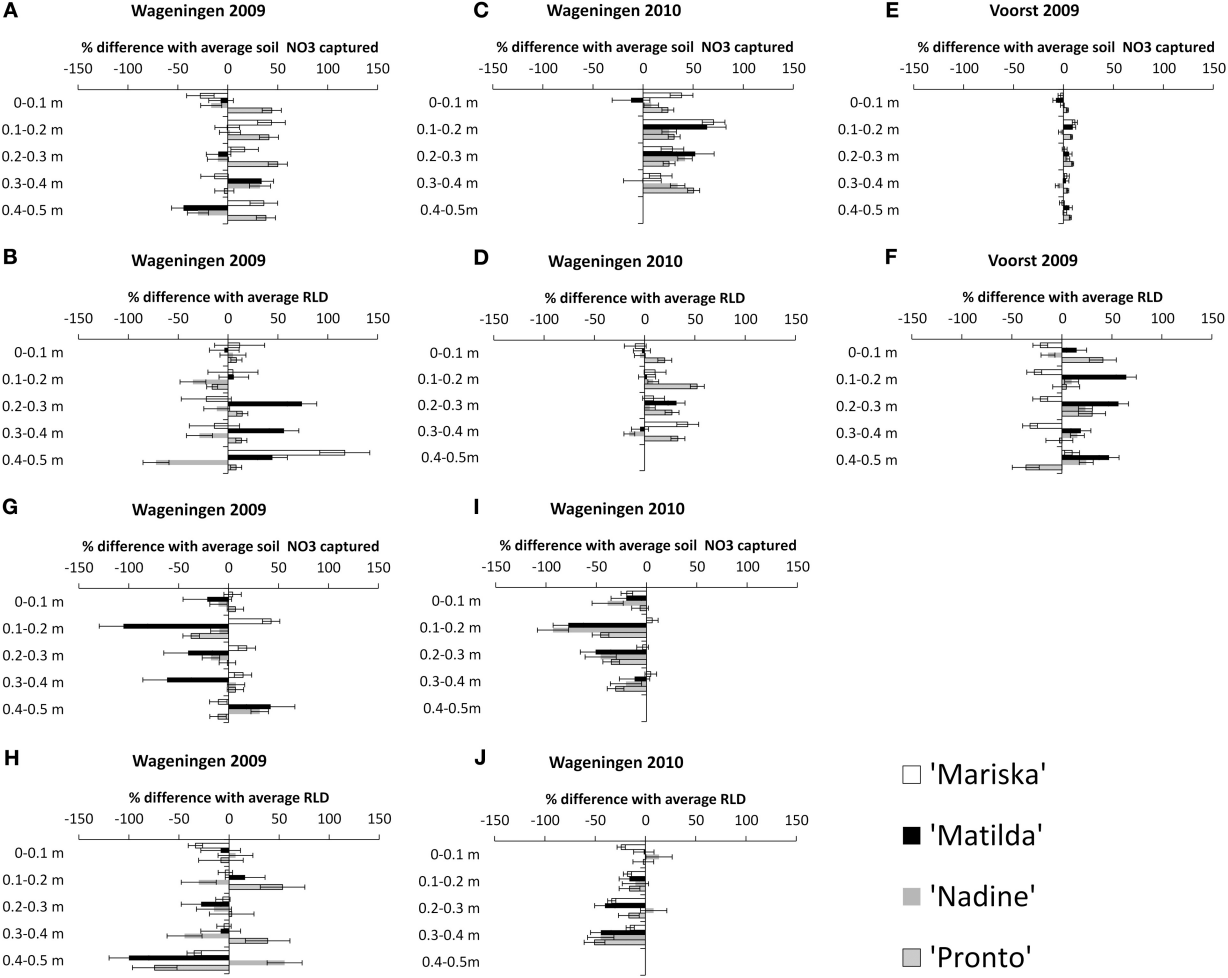
Percentage difference in NO_3_ captured in a layer with the average^*^ NO_3_ captured, and percentage difference in RLD with the average RLD, for each cultivar under the “OD” transplant size (“Over-Developed” transplant size) in trial Wageningen 2009 (A and B), in trial Wageningen 2010 (C and D) and Voorst 2009 (E and F), and under the “UD” transplant size ('Under-Developed' transplant size) in trial Wageningen 2009 (G and H) and Wageningen 2010 (I and J). Error bars indicate ± one standard deviation. ^*^Average and pooled values obtained for all cultivar × transplant size combinations within a layer.

The NO_3_ capture and corresponding root proliferation data are provided in Figure 4. In this figure the percentage difference in Root Length Density (RLD) or in NO_3_ capture between a particular combination of cultivar × transplant size in a given layer, and the average value obtained for the pooled data per layer has been plotted (cf. Materials and Methods). It is surmised that additional RLD is correlated to additional NO_3_ capture in a layer.

***Effect of OD transplant size on NO_3_ capture and root proliferation.*** In Wageningen 2009, no clear pattern emerged showing a higher RLD being proportionally correlated with a higher NO_3_ capture in a layer. Mainly only the OD plants of “Pronto” showed a higher efficiency in NO_3_ capture from the soil in all layers (Figure 4A), but this was not accompanied by a higher RLD than average in these layers (Figure 4B). Conversely, the OD plants of “Matilda” had a higher than average RLD in the 0.3–0.5 m layers but this was not combined with a higher relative NO_3_ capture. “Nadine's” OD plants showed an overall reduced RLD throughout the soil profile. In Wageningen 2009 the correlations were clearer, with an overall higher NO_3_ capture being positively correlated with a slightly higher RLD in all layers for all cultivars (Figures 4C,D). In Voorst 2009, the capture of NO_3_ for the OD plants in all layers did not differ from the average, although the RLD was increased compared with the average for all cultivars through the soil profile, except for “Mariska” (Figures 4E,F).

***Effect of UD transplant size on NO_3_ capture and root proliferation.*** In Wageningen 2009, overall NO_3_ capture was not extremely impaired by a somewhat smaller RLD (Figures 4G,H). “Matilda” showed the most pronounced impaired NO_3_ capture in the 0–0.4 m layers, although this was not associated with a lower RLD in these layers. In Wageningen 2010, NO_3_ capture of UD plants was reduced compared with the average in all layers, and this was well correlated with a reduced RLD throughout the soil profile (Figures 4I,J).

#### Root:shoot ratios over time

Table 6 provides details on the average root:shoot ratios of the four cultivars at the three root sampling dates. Over time the root:shoot ratios declined in all experiments and during the entire period of measurement, except in Wageningen 2009 between the first and second sampling, associated with the low temperatures during the initial growth period in that experiment. Plants in the Voorst 2009 experiment had considerably lower root:shoot ratios than plants in the Wageningen 2009 and Wageningen 2010 experiments at all samplings, in line with the very low root mass observed in the Voorst 2009 experiment. Differences in root:shoot ratios between transplant sizes were only observed in the Voorst 2009 experiment at the second sampling: the normally developed transplants had a higher root:shoot ratio than the over-developed transplants in all cultivars. The same trend was also visible at the first sampling but could not be proven statistically. This general lack of treatment effect even at early stages shows how short-lived the effect of root damage associated with the transplanting actually was and how plastic dry matter partitioning over roots and shoots can be. Significant differences in root:shoot ratio amongst cultivars were found at later sampling dates, but were not always consistent across experiments and were not repeatable over samplings. However, “Pronto” showed consistently high values and “Mariska” consistently low values when cultivar differences proved significant (Table 6).

**Table 6:** **Average root:shoot ratios of the four cultivars at first, second and third root sampling, after establishment from three different transplant sizes in each of three trials**.

**Harvest date**	**CDD^f^ (°Cd)**	**TS^h^**	**Mariska**	**Matilda**	**Nadine**	**Pronto**	
**FIRST ROOT SAMPLING**							
			**Wageningen 2009**				***Tr.*^e^**
April 15th, 2009	111	OD^a^	0.107 ± 0.082^g^	0.113 ± 0.129	0.091 ± 0.056	0.133 ± 0.035	*0.111*a
		ND^b^	0.115 ± 0.051	0.133 ± 0.091	0.072 ± 0.023	0.114 ± 0.038	*0.108*a
		UD^c^	0.095 ± 0.041	0.083 ± 0.059	0.061 ± 0.023	0.073 ± 0.040	*0.078*a
		*Cv.^d^*	*0.105*a^i^	*0.110*a	*0.075*a	*0.107*a	
			**Wageningen 2010**				***Tr*.**
April 26th, 2010	152	OD	0.111 ± 0.106	0.089 ± 0.025	0.114 ± 0.041	0.086 ± 0.022	*0.100*a
		ND	0.094 ± 0.057	0.121 ± 0.046	0.108 ± 0.088	0.071 ± 0.036	*0.099*a
		UD	0.087 ± 0.047	0.087 ± 0.048	0.095 ± 0.055	0.091 ± 0.037	*0.090*a
		*Cv*.	*0.097*a	*0.099*a	*0.106*a	*0.083*a	
			**Voorst 2009**				***Tr*.**
June 8th, 2009	152	OD	0.070 ± 0.029	0.078 ± 0.041	0.072 ± 0.041	0.069 ± 0.041	*0.072*a
		ND	0.070 ± 0.047	0.127 ± 0.162	0.072 ± 0.035	0.071 ± 0.050	*0.085*a
		UD	–	–	–	–	*–*
		*Cv*.	*0.070*a	*0.102*a	*0.072*a	*0.070*a	
**SECOND ROOT SAMPLING**							
			**Wageningen 2009**				***Tr***
April 28th, 2009	224	OD	0.082 ± 0.049	0.102 ± 0.060	0.099 ± 0.040	0.130 ± 0.043	*0.103*a
		ND	0.110 ± 0.022	0.109 ± 0.041	0.084 ± 0.033	0.082 ± 0.040	*0.096*a
		UD	0.086 ± 0.019	0.132 ± 0.049	0.103 ± 0.037	0.128 ± 0.058	*0.112*a
		*Cv*.	*0.093*a	*0.115*a	*0.095*a	*0.113*a	
			**Wageningen 2010**				***Tr*.**
May 10th, 2010	252	OD	0.039 ± 0.012	0.035 ± 0.007	0.048 ± 0.017	0.052 ± 0.026	*0.043*a
		ND	0.043 ± 0.018	0.029 ± 0.010	0.043 ± 0.013	0.063 ± 0.034	*0.045*a
		UD	0.049 ± 0.018	0.043 ± 0.009	0.062 ± 0.017	0.057 ± 0.018	*0.053*a
		*Cv*.	*0.044*ab	*0.036*a	*0.051*bc	*0.058*c	
			**Voorst 2009**				***Tr*.**
June 17th, 2009	253	OD	0.010 ± 0.007	0.012 ± 0.007	0.012 ± 0.006	0.022 ± 0.011	*0.014*a
		ND	0.017 ± 0.012	0.028 ± 0.033	0.021 ± 0.019	0.029 ± 0.019	*0.024*b
		UD	–	–	–	–	*–*
		*Cv*.	*0.014*a	*0.020*a	*0.016*a	*0.025*a	
**THIRD ROOT SAMPLING**							
			**Wageningen 2009**				***Tr*.**
May 11th, 2009	325	OD	0.028 ± 0.011	0.038 ± 0.027	0.033 ± 0.008	0.040 ± 0.019	*0.035*a
		ND	0.026 ± 0.011	0.044 ± 0.021	0.034 ± 0.014	0.045 ± 0.024	*0.037*a
		UD	0.028 ± 0.007	0.042 ± 0.017	0.042 ± 0.017	0.037 ± 0.015	*0.037*a
		*Cv*.	*0.028*a	*0.041*b	*0.036*ab	*0.040*ab	
			**Wageningen 2010**				***Tr*.**
May 24th, 2010	347	OD	0.029 ± 0.011	0.024 ± 0.006	0.021 ± 0.008	0.026 ± 0.009	*0.025*a
		ND	0.023 ± 0.006	0.026 ± 0.011	0.028 ± 0.014	0.029 ± 0.009	*0.027*a
		UD	0.021 ± 0.005	0.025 ± 0.010	0.030 ± 0.012	0.032 ± 0.014	*0.027*a
		*Cv*.	*0.024*a	*0.025*a	*0.026*a	*0.029*a	
			**Voorst 2009**				***Tr*.**
June 29th, 2009	420	OD	0.009 ± 0.002	0.012 ± 0.003	0.012 ± 0.007	0.013 ± 0.004	*0.012*a
		ND	0.009 ± 0.007	0.014 ± 0.007	0.010 ± 0.003	0.019 ± 0.007	*0.013*a
		UD	–	–	–	–	
		*Cv*.	*0.009*a	*0.013*b	*0.011*ab	*0.016*c	

a “Over-developed” transplant size.

b “Normally developed” transplant size.

c “Under-developed” transplant size.

d Mean for cultivar across transplant sizes.

e Mean for transplant size across cultivars.

f Cumulated Degree-Days.

g Standard error of the mean.

h Transplant Size.

i Means with different letters indicate a significant difference at p ≤ 0.05—means separation with lettering is within an experiment and at the level of main factors cultivar or transplant size when the two-way interaction was not significant and at the level of transplant size × cultivar when the interaction was significant.

#### Physiological nitrogen use efficiency (NUE) and nutritional status of the plant

***Physiological nitrogen use efficiency.*** Significant interactions were found between transplant sizes and cultivar effects on physiological NUE (defined as g dry weight per g nitrogen found in the plant) in Wageningen 2010 and Voorst 2009 (Table 7). In Wageningen 2009, OD and UD plants had a significantly reduced physiological NUE compared to ND plants. Overall, “Nadine” showed to have a higher physiological NUE whatever transplant size was applied, compared to “Mariska.” In Wageningen 2009, this cultivar had the lowest physiological NUE. In Wageningen 2010, OD and ND plants of “Matilda” had a significantly higher physiological NUE than OD plants of “Mariska.”

**Table 7:** **Average physiological NUE (g DM g^−1^ N in head) of the four cultivars at final harvest, after establishment from three different transplant sizes**.

**Harvest date**	**CDD^f^ (°Cd)**	**TS^h^**	**Mariska**	**Matilda**	**Nadine**	**Pronto**	
			**Wageningen 2009**				***Tr.*^e^**
May 25th, 2009	474	OD^a^	29.1 ± 1.8^g^	29.6 ± 1.9	32.7 ± 3.0	31.3 ± 3.6	*30.7a*
		ND^b^	30.6 ± 1.5	33.0 ± 4.5	33.1 ± 2.5	32.0 ± 2.3	*32.2b*
		UD^c^	30.3 ± 1.3	30.2 ± 2.7	31.8 ± 1.8	29.3 ± 1.5	*30.4a*
		*Cv.^d^*	*30.0*a^i^	*31.0*ab	*32.5*b	*30.9*ab	
			**Wageningen 2010**				***Tr*.**
May 30th, 2010	400	OD	34.8 ± 4.2a	45.1 ± 4.9d	41.7 ± 3.0cd	40.5 ± 3.0abcd	*40.5*
		ND	39.2 ± 6.2abcd	44.8 ± 3.8d	41.5 ± 3.8bcd	38.4 ± 2.4abc	*41.0*
		UD	35.5 ± 3.4ab	37.1 ± 2.8abc	39.7 ± 2.2abcd	36.6 ± 2.2abc	*37.2*
		*Cv*.	*36.5*	*42.3*	*41.0*	*38.5*	
			**Voorst 2009**				***Tr*.**
June 29th, 2009	420	OD	24.9 ± 1.8ab	24.1 ± 0.6ab	24.6 ± 0.6ab	24.2 ± 0.6ab	*24.5*
		ND	23.1 ± 0.8a	22.8 ± 1.0a	25.6 ± 3.0b	23.0 ± 0.7a	*23.6*
		UD	–	–	–	–	
		*Cv*.	*24.0*	*23.4*	*25.1*	*23.6*	

a “Over-developed” transplant size.

b “Normally developed” transplant size.

c “Under-developed” transplant size.

d Mean for cultivar across transplant sizes.

e Mean for transplant size across cultivars.

f Cumulated Degree-Days.

g Standard error of the mean.

h Transplant Size.

i Means with different letters indicate a significant difference at p ≤ 0.05—means separation with lettering is within an experiment and at the level of main factors cultivar or transplant size when the two-way interaction was not significant and at the level of transplant size × cultivar when the interaction was significant.

In Voorst 2009, physiological NUE values were lower than values obtained for the Wageningen trials (Table 7). No significant difference in physiological NUE values was found between transplant sizes or between cultivars. Only within the ND plants, “Nadine” had a significantly higher physiological NUE than the other cultivars.

**Figure 5:**
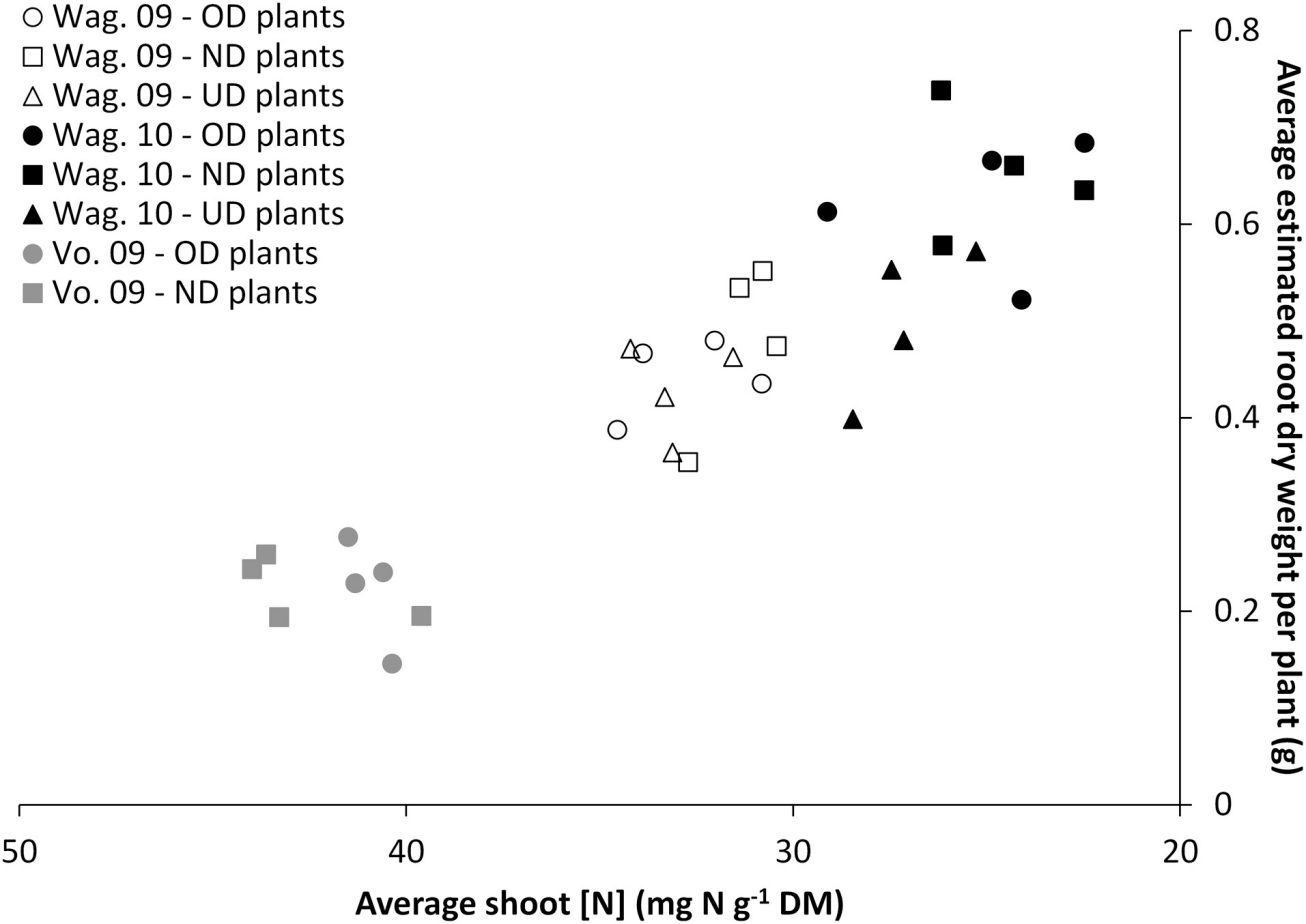
**Relationship between the nutritional status of the plant (average shoot [N]) and its estimated root weight for the two or three transplant sizes [Over-Developed- (“OD”), Normally Developed- (“ND”) and Under-Developed- (“UD”) transplant size], measured at the final harvest in the trials Wageningen 2009 (“Wag. 09”), Wageningen 2010 (“Wag. 10”) and Voorst 2009 (“Vo. 09”)**.

***Nutritional status of the plant.*** Figure 5 shows the relationship between the nutritional status of the plant (shoot [N]) and its estimated root dry weight for the three trials at the respective final harvests. The alignment of the data obtained for the three trials highlights that the final harvests took place at different nutritional statuses of the plants which were proportionally related to root dry weight.

## Discussion

Transplanting four cultivars at three different transplant stages gave a significant insight into the impact of below-ground physiological processes developed by lettuce to overcome the short-lived stresses created by altering the initial root:shoot ratio and to maintain shoot growth. Strong Treatment × Environment interactions were visible in these trials.

### Seasons and soil conditions impacted transplant size effect on shoot and root growth: treatment X environment interactions

The early spring growing seasons in the Wageningen 2009 and 2010 trials were to a certain extent similar in terms of photoperiod and soil conditions (texture, CEC, etc.) although the Wageningen 2010 trial experienced slightly more rainfall (Table 1) and a colder start (cf. Materials and Methods) than the Wageningen 2009 trial; in contrast, the Voorst 2009 trial was conducted later in the season, under higher soil and air temperatures and likely higher levels of radiation (not recorded), which led to much higher relative growth rates during the initial growth phase (Table 2). On the other hand, whereas maximal growth rates during the linear phase reached average values between 100 (Wageningen 2009) and 130 (Wageningen 2010) mg DM m^−2^ (°Cd)^−1^, these rates remained below 100 mg DM m^−2^ (°Cd)^−1^ for Voorst 2009 (Table 2). This influenced the effects of transplant sizes to a large extent, as the differences between the OD and the ND plants were significant in the Voorst 2009 trial but not in the early spring trials in Wageningen (Table 3). In Voorst 2009, the warm growing conditions even led to failure of UD plants, of which head formation and maturation did not occur within the time frame of the experiment, despite the higher cumulated thermal time.

Figure 5 shows that the root dry weight of the plants under the various transplant sizes was not driven by the transplant size and/or the cultivars, but rather a function of the nutritional status i.e., the growth stage. The higher shoot N concentration for some treatments is an indication of physiologically younger plants. Here shoot N is diluted over less biomass as shown by the smaller dry weights. Comparison of these data with the root:shoot ratio and shoot dry weight data in Tables 7 and 3, respectively shows that the harvested plants at the lower shoot N concentration also had a higher root:shoot ratio. This may have been related to the functional equilibrium change under reduced plant nitrogen status (Poorter and Nagel, 2000).

### Unbalanced root:shoot ratio created by root pruning at transplanting has short-lasting effects on shoot growth

Root pruning at transplanting using overdeveloped seedlings did not impact the yield at final harvest in the Wageningen trials (Table 3). The mechanical damage inflicted to the roots of the OD plants at transplanting did not impact root growth either, as no significant difference was found between the OD and the ND plants in total root weight at any sampling date or in RLD at any soil depth for any sampling date (data not shown). Any impact of the treatment on the root:shoot ratios had already disappeared at first sampling in the Wageningen experiments and only showed itself temporarily in Voorst 2009 (Table 6). For the three trials, OD plants showed an overall lower maximal relative growth rate (Table 2) and an overall lower maximal leaf expansion rate (Table 4) during the exponential phase compared with the ND plants, which was caused by their bigger size at transplanting compared to ND plants (therefore a lower amount of tissue produced per amount of existing tissue in the exponential phase). However, this did not influence the start of the linear growth phase, as no significant difference in lag phase was found for dry weight accumulation (Table 2) or leaf expansion (Table 4), except in Wageningen 2009. These results suggest that for the lettuce cultivars used in this study, a mild root pruning at transplanting is not a large stress for shoot growth and does not affect final yield in the early spring season. The moderate soil and air temperatures, light intensity and radiation (not recorded) in the Wageningen trials led to a slower shoot growth, especially in the exponential phase (Table 2), and consequently required less from the roots to sustain the growth. This may explain why the stress created by root pruning was not crucial for shoot growth for these trials. In contrast, the higher air and soil temperatures recorded in the Voorst trial (late spring/early summer) increased the shoot growth rates in the exponential phase (Table 2) and emphasized the important role of a larger root system in this trial to sustain the growth of larger shoots such as the OD plants. This was very visible in the results, as the cultivars with the largest root weight (“Matilda” and “Pronto,” Table 5) under both the OD and the ND transplant size, performed better in terms of shoot weight (Table 3) than “Mariska” which had the smallest root weight (Table 5).

### Transplanting underdeveloped plants impacts root and shoot growth to a large extent

Transplanting UD seedlings in open field conditions imposes considerable physiological stress on growth and development of the plant. UD plants were not able to recover from transplant shock and to catch up with ND plants during the experiments in terms of dry weight accumulation, especially for Wageningen 2010 (Supplementary materials, Tables S1, S2 and Table 3). Vos et al. (1996) showed that leaf initiation and potential leaf size are largely determined before leaves actually appear, i.e., the number of leaves and the size of the leaves are determined already in the apex. They hypothesized that stress at an early growth stage may disturb the physiological mechanisms controlling leaf initiation in the apex, and may therefore affect later field performance over a longer time, as observed in our experiments. The smaller size at transplanting impacted shoot growth: the UD plants' smaller leaf area at transplanting increased the maximal relative growth rate/leaf expansion rate during the exponential phase (Table 2) which increased the lag phase, as the UD plants required more time to finalize the exponential growth period. As a result UD plants had slightly smaller heads and delayed maturity (data not shown). In practice, transplanting smaller plants, delaying maturity, translates into a longer period in the field and consequently some financial loss for the grower.

The transplanting shock did not only affect shoot growth and development. We surmise that the shock imposed on the plants by transplanting underdeveloped seedlings also disturbs root initiation and leads to a smaller root system for the UD plants compared to the ND plants, as observed in Wageningen 2010 (Table 5), the trial with lowest soil temperatures. The smaller root system was not compensated by an improved NO_3_ capture capacity, as shown clearly for Wageningen 2010 in Figure 4.

### Genetic variation in root:shoot growth strategies

The four cultivars were chosen according to their different growth patterns in the field as well as their specific root mass distributions over the soil profile as observed previously by Den Otter and Lammerts van Bueren (2007). The diverse strategies exhibited by the cultivars to overcome the transplant shock seemed rather consistent across years.

“Mariska” was a cultivar which had the smaller root system overall (Figures 5A,E,I). For this cultivar, root pruning tended to increase total root mass consistently in Wageningen 2009 and 2010 (Table 5) which underlines a powerful root regeneration capacity. In practice, the cultivar Mariska is often preferred for the early spring growing season, when weather conditions force growers to delay the planned planting date. They are then faced with overdeveloped transplants, a situation from which the cultivar is known to recover easily (K. de Jong, pers. commun.). This research shows that for “Mariska” this high root regeneration capacity is however a trade-off for shoot growth, as the larger assimilate allocation to the roots was at the expense of the shoot, which tended to be lighter than that of the other cultivars at final harvest (Table 3).

In contrast to “Mariska,” “Matilda,” and “Pronto” were the two cultivars which had the largest root system (Table 5), whereas Pronto often had the highest root:shoot ratio (Table 6). Such a large root system may have contributed to their steady good field performance across transplant size, locations, and years (Table 3); indeed developing more roots, especially in deeper soil layers (as it was measured for these cultivars in layers 0.1–0.2 and 0.3–0.4 m, Figures 3B,F,J for “Matilda” and Figures 3D,F,L for “Pronto”) increased resource capture quantitatively and consequently conferred a proportional advantage for shoot performance. Besides, the results of this study suggest that these cultivars are relatively robust, as their response to transplant shock (either root pruning or underdeveloped transplant size) was consistent over locations and seasons. In practice, these cultivars are often preferred by “hobby” gardeners as robust cultivars when growing conditions are less controlled and less optimal, which confirms our findings. However, it must be underlined that the field conditions under which the trials were carried out in this study were rather optimal, as no strong drought or nitrate leaching occurred. It might be that a larger proportion of assimilates allocated to root proliferation as displayed by “Matilda” and “Pronto” could be a trade-off for final yield in case of less optimal field conditions, e.g., temporary drought or spatial limitation in nitrate availability. Other physiological mechanisms involved in nitrate capture e.g., improved nitrate inflow per unit root length (Vuuren et al., 1996) may then confer robustness.

Finally, “Nadine” is a cultivar that had a relatively smaller root system but had a higher physiological NUE than the other cultivars (Table 7). This cultivar performed consistently in all three experiments under all transplant sizes, underlining the fact that not only the capacity to take up resources from the soil is important, but also the internal ability to use these resources in order to ensure adequate shoot growth despite environmental stresses.

## Concluding remarks

This study investigated the effect of different types of transplant shocks, created by root pruning or underdeveloped transplant size, on field performance of lettuce, and the role of below-ground traits in overcoming such disturbances. The results of three field experiments showed that the mechanical damage inflicted at transplanting to the roots of overdeveloped transplants has short-lasting effects on shoot growth and does not impact final yield. This suggests that the plants respond quickly to such a shock by adaptive responses at the root level, and are able to restore the initial root:shoot ratio fast enough not to impact final yield. Strategies to overcome the mechanical damage at the root level include high root regeneration capacity, which however, can be trade-off for shoot yield as shown for “Mariska.”

On the other hand, a large transplant shock, created by transplanting underdeveloped seedlings, cannot be overcome by lettuce; the results showed that transplanting undeveloped seedlings has lasting effects on overall root and shoot growth: slower growth results in smaller plants that mature later.

Overall, more roots in deeper layers, as observed for “Matilda” and “Pronto,” was linked to stable field performance despite transplant shock across trials, locations, and seasons, and may therefore constitute a trait of robustness for lettuce, as we hypothesized. If a more developed root system enables the plants to sustain growth during temporary periods of drought or nitrate shortage by capturing resources from deeper soil layers, the ability to efficiently transform the captured resources into shoot mass is also an important trait for robustness, as found for “Nadine” in these trials.

Monitoring spatial and temporal changes in below-ground cues and measuring their effects on above-ground parameters were only feasible in this study by using a limited set of cultivars, selected on the basis of specific criteria. In no way do we suggest that our results are fully representative for the genetic variation present among the numerous lettuce varieties. Instead, this study, together with a previous paper reporting on the spatial and temporal dynamics of root development and resource capture in lettuce (Kerbiriou et al., 2013), will provide the basis for a conceptual framework to design a strategy to breed lettuce for robustness, which will be used to interpret results obtained from a large set of lettuce varieties trialed in diverse environmental conditions.

### Conflict of interest statement

The authors declare that the research was conducted in the absence of any commercial or financial relationships that could be construed as a potential conflict of interest.

## Supplementary material

The Supplementary Material (Tables S1, S2, S3, S4) for this article can be found online at: http://www.frontiersin.org/Functional_Plant_Ecology/10.3389/fpls.2013.00379/abstract
