# Supplementary material for: Influence of transplant size on the above- and below-ground performance of four contrasting field-grown lettuce cultivars
Source: Front Plant Sci. 2013 Sep 27;4:379. doi: 10.3389/fpls.2013.00379 (PMC3784774; doi:10.3389/fpls.2013.00379)
Supplement: Supplementary file 1 [file 51691_Kerbiriou_DataSheet1.PDF]

**Supplementary material**

**Table S1. Average shoot dry weights (g per plant) of the four cultivars at first root sampling, after establishment from three different transplant sizes in each of three trials.**

| Harvest Date | CDD[6] (ºCd) | TS[8] | Mariska | Matilda | Nadine | Pronto | |
|---|---|---|---|---|---|---|---|
| | | | **Wageningen 2009** | | | | *Tr.*[5] |
| April 15th, 2009 | 111 | OD[1] | 0.58±0.08[7] | 0.62±0.05 | 0.49±0.06 | 0.57±0.15 | *0.56*c |
| | | ND[2] | 0.47±0.05 | 0.45±0.05 | 0.46±0.11 | 0.48±0.05 | *0.46*b |
| | | UD[3] | 0.30±0.03 | 0.24±0.06 | 0.27±0.04 | 0.32±0.04 | *0.28*a |
| | | *Cv.*[4] | *0.45*a[9] | *0.44*a | *0.41*a | *0.45*a | |
| | | | **Wageningen 2010** | | | | *Tr.* |
| April 26th, 2010 | 152 | OD | 2.48±0.31 | 2.43±0.44 | 2.93±0.37 | 2.69±0.26 | *2.63*c |
| | | ND | 2.28±0.41 | 2.04±0.51 | 2.11±0.29 | 2.42±0.46 | *2.21*b |
| | | UD | 1.18±0.28 | 0.79±0.11 | 0.99±0.15 | 0.93±0.41 | *0.97*a |
| | | *Cv.* | *1.98*a | *1.75*a | *2.01*a | *2.02*a | |
| | | | **Voorst 2009** | | | | *Tr.* |
| June 8th, 2009 | 152 | OD | 1.46±0.30 | 1.10±0.42 | 1.48±0.36 | 1.34±0.28 | *1.34*b |
| | | ND | 0.51±0.10 | 0.30±0.20 | 0.36±0.07 | 0.49±0.09 | *0.43*a |
| | | UD | - | - | - | - | |
| | | *Cv.* | *0.99*b | *0.73*a | *0.92*ab | *0.91*ab | |

[1]'Over-developed' transplant size; [2]'Normally developed' transplant size; [3]'Under-developed' transplant size; [4]Mean for cultivar across transplant sizes; [5]Mean for transplant size across cultivars; [6]Cumulated Degree-Days; [7]Standard error of the mean; [8]Transplant Size; [9]Means with different letters indicate a significant difference at $p \leq 0.05$ – means separation with lettering is within an experiment and at the level of main factors cultivar or transplant size when the two-way interaction was not significant and at the level of transplant size × cultivar when the interaction was significant.
