# Supplementary material for: Influence of transplant size on the above- and below-ground performance of four contrasting field-grown lettuce cultivars
Source: Front Plant Sci. 2013 Sep 27;4:379. doi: 10.3389/fpls.2013.00379 (PMC3784774; doi:10.3389/fpls.2013.00379)
Supplement: Supplementary file 2 [file 51691_Kerbiriou_DataSheet2.PDF]

Table S2. Average shoot dry weights (g per plant) of the four cultivars at second root sampling, after establishment from three different transplant sizes in each of three trials.

Harvest Date	CDD[6] (ºCd)	TS[8]	Mariska	Matilda	Nadine	Pronto	
			**Wageningen 2009**				*Tr.*[5]
April 28th, 2009	224	OD[1]	3.83±0.35[7]	3.23±0.74	3.41±0.47	3.33±0.39	*3.45*b
		ND[2]	3.52±0.47	2.95±0.26	3.49±0.57	3.36±0.47	*3.33*b
		UD[3]	3.06±0.83	2.37±0.35	2.44±0.28	2.71±0.49	*2.64*a
		Cv.[4]	*3.47*b[9]	*2.85*a	*3.11*a	*3.13*ab	
			**Wageningen 2010**				*Tr.*
May 10th, 2010	252	OD	8.58±1.65	8.67±1.18	8.14±1.14	8.20±2.06	*8.40*b
		ND	7.98±1.18	8.78±1.42	7.83±1.03	7.17±1.39	*7.94*b
		UD	4.72±0.82	4.54±0.95	4.19±1.31	4.57±1.00	*4.50*a
		Cv.	*7.09*a	*7.33*a	*6.72*a	*6.65*a	
			**Voorst 2009**				*Tr.*
June 17th, 2009	253	OD	10.55±1.27	8.55±1.08	11.23±1.88	8.19±2.02	*9.63*b
		ND	5.80±1.92	3.80±1.45	3.14±0.61	3.45±0.70	*4.05*a
		UD	-	-	-	-	
		Cv.	*8.18*c	*6.18*ab	*7.18*bc	*5.82*a	

[1]'Over-developed' transplant size; [2]'Normally developed' transplant size; [3]'Under-developed' transplant size; [4]Mean for cultivar across transplant sizes; [5]Mean for transplant size across cultivars; [6]Cumulated Degree-Days; [7]Standard error of the mean; [8]Transplant Size; [9]Means with different letters indicate a significant difference at $p \leq 0.05$ – means separation with lettering is within an experiment and at the level of main factors cultivar or transplant size when the two-way interaction was not significant and at the level of transplant size × cultivar when the interaction was significant.
