# Supplementary material for: Influence of transplant size on the above- and below-ground performance of four contrasting field-grown lettuce cultivars
Source: Front Plant Sci. 2013 Sep 27;4:379. doi: 10.3389/fpls.2013.00379 (PMC3784774; doi:10.3389/fpls.2013.00379)
Supplement: Supplementary file 3 [file 51691_Kerbiriou_DataSheet3.PDF]

Table S3. Average estimated root dry weights (g per plant) of the four cultivars at first root sampling, after establishment from three different transplant sizes in each of three trials.

Harvest Date	CDD[6] (ºCd)	TS[8]	Mariska	Matilda	Nadine	Pronto	
			**Wageningen 2009**				*Tr.*[5]
April 15th, 2009	111	OD[1]	0.06±0.05[7]	0.07±0.09	0.04±0.03	0.07±0.03	*0.06*b
		ND[2]	0.05±0.02	0.06±0.05	0.03±0.01	0.05±0.01	*0.05*b
		UD[3]	0.03±0.01	0.02±0.01	0.02±0.01	0.02±0.01	*0.02*a
		Cv.[4]	*0.05*a[9]	*0.05*a	*0.03*a	*0.05*a	
			**Wageningen 2010**				*Tr.*
April 26th, 2010	152	OD	0.30±0.34	0.21±0.06	0.34±0.13	0.23±0.06	*0.27*b
		ND	0.21±0.14	0.24±0.13	0.23±0.20	0.17±0.08	*0.21*b
		UD	0.10±0.04	0.07±0.04	0.09±0.05	0.09±0.05	*0.09*a
		Cv.	*0.20*a	*0.18*a	*0.22*a	*0.16*a	
			**Voorst 2009**				*Tr.*
June 8th, 2009	152	OD	0.09±0.02	0.08±0.06	0.10±0.05	0.08±0.03	*0.09*b
		ND	0.04±0.03	0.03±0.02	0.02±0.01	0.03±0.02	*0.03*a
		UD	-	-	-	-	
		Cv.	*0.07*a	*0.06*a	*0.06*a	*0.06*a	

[1]'Over-developed' transplant size; [2]'Normally developed' transplant size; [3]'Under-developed' transplant size; [4]Mean for cultivar across transplant sizes; [5]Mean for transplant size across cultivars; [6]Cumulated Degree-Days; [7]Standard error of the mean; [8]Transplant Size; [9]Means with different letters indicate a significant difference at $p \leq 0.05$ – means separation with lettering is within an experiment and at the level of main factors cultivar or transplant size when the two-way interaction was not significant and at the level of transplant size × cultivar when the interaction was significant.
